# Towards more efficient synthetic immunomodulators: biological characterization and mechanism of action of monosaccharide-derived TLR4 agonists†

**DOI:** 10.1039/d4md00950a

**Published:** 2025-02-18

**Authors:** Ana Rita Franco, Zaineh Aladailleh, Alessio Romerio, Alice Italia, Federico Lami, Mohammed Monsoor Shaik, Natalia Skupinska, Valentina Artusa, Grisha Pirianov, Francesco Peri

**Affiliations:** a Department of Biotechnology and Biosciences, University of Milano-Bicocca Piazza della Scienza, 2 20154 Milano (MI) Italy francesco.peri@unimib.it; b School of Life Sciences, Anglia Ruskin University East Road Cambridge CB1 1PT UK

## Abstract

Toll-like receptors (TLRs), including TLR4, play a crucial role in innate immunity activation, and small molecular TLR4 activators (agonists) are in the preclinical and clinical phases of development as vaccine adjuvants or tumor immunotherapeutics. Recently, we generated novel glucosamine-derived compounds, FP molecules, that are active as TLR4 agonists. Despite their chemical structure differing from LPS, some of these compounds, including compound FP18, mimicked the biological activity of LPS and its capacity to activate TLR4 and its downstream pathways. In contrast to FP18, compound FP20 showed immunostimulant activity that was only partially due to TLR4 agonism. This activity was mainly associated with NLRP3 inflammasome activation. We generated a panel of glycosylated FP20 derivatives (glyco-FP20) bearing different monosaccharides linked to C6 of the glucosamine. The biological activity of glyco-FP20 was related to TLR4 activation, as assessed from preliminary experiments in HEK-Blue cells. We presented a comprehensive study of the mechanism of action of glyco-FP20 derivatives and their effect on TLR4 signalling, leading to macrophage M1 polarisation and pyroptosis in THP-1 derived macrophages. Results revealed that, similarly to LPS and differently from FP20, glyco-FP20 derivatives were potent TLR4 agonists inducing TLR4/MyD88 signalling pathways that led to M1 macrophage polarisation, associated with NF-kB activation/translocation and release of a number of proinflammatory mediators in THP-1-derived macrophages. In particular, compound FP20 Rha activated TLR4/TRIF signalling, associated with phosphorylation of STAT1/IRF3, leading to the production of IFN-β in THP-1-derived macrophages. Furthermore, using a specific GSD inhibitor (U73), we demonstrated the ability of FP20 and glyco-FP20 to induce GSD-dependent pyroptosis, which was associated with IL-1β/IL-18 and LDH release in THP-1-derived macrophages. These results show that the optimization of FP20 glycosylation can increase the biological potency of the parent molecule and can be used in preclinical development as vaccine adjuvants or macrophage-based cancer immunotherapy.

## Abstract 2

The new synthetic glycosylated FP20 derivatives strongly activate TLR4 pathways, namely the MyD88 and the TRIF/IRF3 pathways with subsequent cytokine and IFN-β production and they induce GSD-dependent pyroptosis.

## Graphical abstract

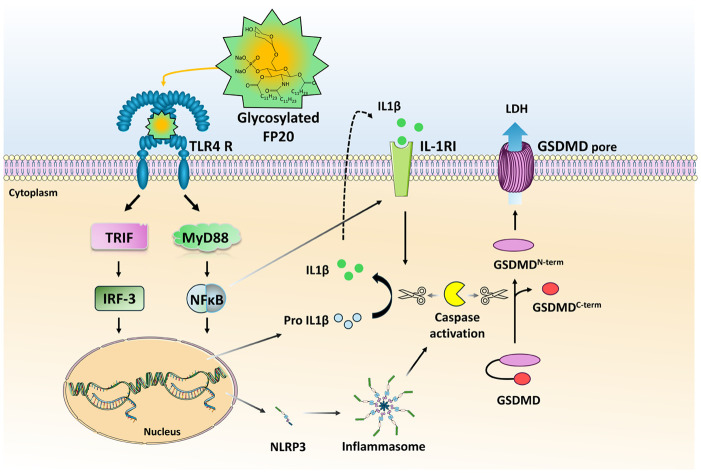

## Introduction

The development of small molecules capable of stimulating innate and adaptive immunity with defined mechanisms of action is highly relevant for the development of effective immune-based therapeutics. An important class of molecules in the preclinical and clinical development phases as vaccine adjuvants and tumour immunotherapeutics are compounds that stimulate the innate immunity receptor TLR4 (toll-like receptor 4), thus mimicking the action of endotoxin, namely, lipopolysaccharide (LPS), the natural TLR4 agonist. Lipopolysaccharide (LPS), the major component of the outer membrane of Gram-negative bacteria, binds the toll-like receptor 4 (TLR4)–MD2 complex and activates innate immune responses. LPS transfer to TLR4–MD2 is catalysed by both LPS binding protein (LBP) and CD14.^1^ LPS consists of lipid A, core oligosaccharide, and O-antigen. Multiple acyl chains and two phosphate groups of lipid A are critical for the M-shaped dimerization of the TLR4–MD2 complex.^2^ Binding of LPS leads to the initiation of MyD88- and TRIF-dependent signal cascades.^3^ The driving force of TLR4 activation by endotoxin is the interaction of lipid A lipid chains and the disaccharide moiety with MD-2. The lipid chains are completely buried in the MD-2 cavity, with only one chain (R2) partially exposed to the MD-2 surface, composing the core hydrophobic interface for interaction with the second TLR4 of the dimer, called TLR4.^2^ The ester and amide groups connecting the lipids to the glucosamine backbone or to the other lipid chains are exposed to the surface of MD-2. They interact with hydrophilic side chains located on the beta strand of the MD-2 pocket and on the surface of TLR4 and the second TLR4 in the dimer. The two phosphate groups of lipid A bind to the TLR4–MD-2 complex by interacting with the positively charged residues in TLR4, TLR4* (the second TLR4 unit of the dimer) and MD-2, forming a hydrogen bond with S118 of MD-2. Because of the absence of one of these phosphates, the lipid A mimetic monophosphoryl lipid A (MPLA) presents reduced toxicity compared to lipid A due to its weaker binding with TLR4/MD-2. However, MPLA can activate TLR4 with a bias towards the TRIF/IRF3 pathway, which is currently used as a vaccine adjuvant.^4^ Both hydrophobic and hydrophilic interactions contribute to the main dimerization interaction between MD-2/LPS and TLR4. The carbohydrates in the inner core of LPS form several bonds with metal ions and the MD-2 and TLR4 molecules. However, the importance of these carbohydrate interactions in receptor dimerization is not obvious because they are not essential for the endotoxic activity of LPS.

**Fig. 1:**
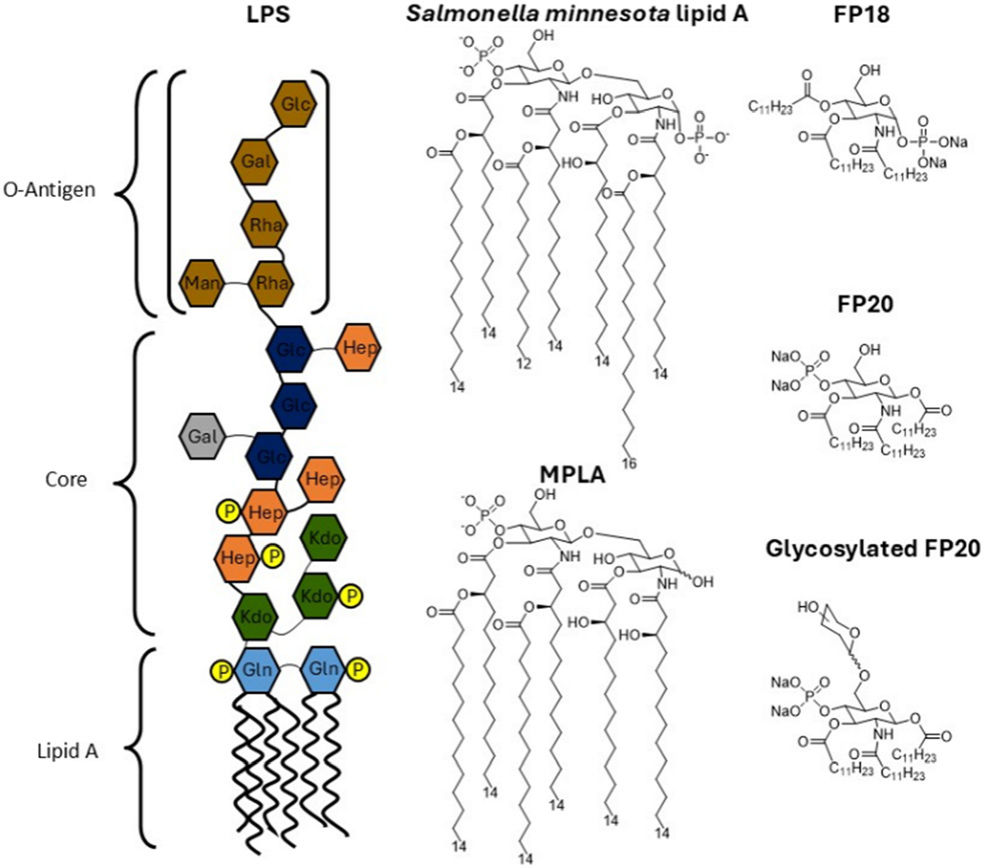
Molecular formula of lipopolysaccharide (LPS), *Salmonella minnesota* lipid A, monophosphoryl lipid A (MPLA) and FP compounds.

Aiming to achieve molecular simplification of lipid A/LPS with the perspective to develop vaccine adjuvants or tumour immunotherapeutics, we developed FP molecules as structurally simplified, monosaccharide-based TLR4 agonists (Fig. 1).^5,6^ Although the FP18 molecule has a very similar activity profile and MOA to LPS and MPLA, and activates the MyD88, TRIF/IRF3 pathway and NLRP3 inflammasomes, FP20 showed selective action on NLRP3 inflammasomes with very weak or no action on the MyD88 and TRIF pathways.^6^ FP20 did not induce NF-κB (p65 subunit) or p-IRF-3 nuclear translocation in the immunofluorescence experiments, while it induced p38 MAPK activation. Given these data, FP20 should be more properly defined an inflammasome activator rather than a TLR4 agonist. Although being less active than FP18, FP20 presents interesting *in vivo* activity. The *in vivo* data with the OVA antigen confirmed the low toxicity of FP20 as well as its immunostimulatory ability, especially after the first immunization.^6^

Subsequently, we aimed to modify the structure of FP20 by adding a sugar unit to C6, thus mimicking the first sugar attached to lipid A in the oligosaccharide core of LPS. The hydrophilic core oligosaccharide of LPS plays an important role by directly interacting with the TLR4/MD-2 receptor complex. This interaction, mainly mediated by the first monosaccharide bound to lipid A (Kdo I), is crucial to increase the binding affinity between LPS and TLR4.^7^ Therefore, the rationale guiding the design of glycosylated FP20 (Fig. 1) is that adding a monosaccharide moiety mimicking Kdo can increase the affinity of FP20 for the receptor and its biological activity. The addition of a sugar unit also improved the water solubility and the bioavailability of the glycosylated derivatives for *in vivo* and *in vitro* studies. This led to a new class of compounds with increased activity on HEK-TLR4 (human TLR4).^8^ FP20 linked to a rhamnose monosaccharide (FP20-Rha) showed ability to activate inflammatory circuits in human macrophages.^9^ In a vaccine study, using ovalbumin (OVA) as the antigen, in C57BL/6 mice, FP20Rha could induce specific anti-OVA IgG with a potency similar to MPLA, while FP20 showed lower adjuvant potency.^9^ Herein, we present the activity of five glycosylated derivatives, bearing glucose, mannose, galactose, lyxose and rhamnose monosaccharides linked through glycosidic bond to the C-6 position of FP20. Subsequently, we investigated the mechanism of action in all the compounds, and in particular in the lead compound bearing rhamnose. Different from the FP20 precursor, glyco-FP20, and in particular the lead compound FP20 Rha, strongly activated both the MyD88 and TRIF pathways, paralleling the behaviour of LPS/lipid A and MPLA. Due to their biological activity, these compounds have potential to be used as vaccine adjuvants in novel vaccine formulations as well as in immunotherapeutics targeting TLR4.

## Experimental

### Materials and methods

The TLR4 agonist glyco-FP20 derivatives were synthesized in the laboratory of Prof. F. Peri (University of Milano-Bicocca) *via* multistep organic synthesis and the purity and identity of the compounds were confirmed by NMR, mass spectrometry and HPLC analyses. In the case of TLR4-exclusive and potent activation, LPS (*Salmonella minnesota* (S-form), TLRpure™) was used (Innaxon Biosciences, Tewkesbury, UK). For the *in vitro* experiments, the glyco-FP20 derivatives were reconstituted in DMSO (5 mM stock).

### Cells maintenance and treatment

THP-1 cells were obtained from the European Collection of Animal Cell Cultures (Salisbury, Wiltshire, U.K.) and cultured in RPMI (+10% heat inactivated foetal bovine serum (HIFBS) + 1% glutamine + 1% penicillin/streptomycin). The cells were split 3 times weekly and maintained at a density of ∼0.5 × 10^6^ cells per mL. For the differentiation of the THP-1 cells, 25 nM of PMA (phorbol-12-myristate 13-acetate) was added to plated cells for 3 days before washing three times with fresh medium. Then, the cells were left to rest overnight before treatment. All the cells were treated with the glyco-FP20 derivatives (0–20 μM) or exposed to LPS (100 ng mL^−1^) for 0 to 16 h.

### MTT assay

The MTT assay was performed to evaluate the effect of the glyco-FP20 derivatives on the viability of THP-1-derived macrophages. The cells were treated with different concentrations of glyco-FP20 derivatives. MTT was dissolved in PBS (5 mg mL^−1^). Subsequently, 25 μL of the MTT solution was added to each well of a 96-well plate, containing 50 000 cells per well. Then, the cells were incubated at 37 °C with 5% CO_2_ for 2 h. A blank control solution was prepared using complete medium without cells. Finally, the cells were solubilised with 10% SDS overnight at 37 °C. The results were read at the absorbance wavelength of 620 nm.

### SEAP cell reporter assay

After the addition of the compounds and selected controls, the cells were incubated for 16 to 18 h. The supernatants were collected, and the SEAP levels were quantified using the QUANTI-Blue assay (InvivoGen). Briefly, 20 μL of supernatant of reporter cells, THP-1 X-Blue-derived macrophages, was incubated with 180 μL of QUANTI-Blue solution in a 96-well plate for 0.5–4 h at room temperature. SEAP activity, as an indicator of TLR4 activation, was assessed by reading the optical density (OD) of the well at 630 nm. The results were normalized with the positive control LPS and expressed as the mean of the percentage ± standard error of the mean (SEM) of at least three independent experiments.

### LDH assay

The effect of the glyco-FP20 derivatives on the cell viability associated with pyroptosis was assessed using the LDH assay. THP-1 cells were seeded in 96-well plates (0.5 × 10^6^ mL^−1^). The cells were differentiated with PMA (25 nM) for 3 days. PMA was removed by washing and the THP-1-derived macrophages were left for 24 h in fresh medium before exposure to the glyco-FP20 derivatives or LPS for a further 24 h. Then 10 μL medium from each sample/well was analysed for LDH release following the manufacturer's instructions (Abcam, UK). Finally, the plates were read at 450/620 nm.

### Western blot analysis of protein expression and phosphorylation

Cell lysates (50 μg) were separated on 7.5% TGX gels, transferred to polyvinyldifluoride membranes (Bio-Rad, UK) and blocked using 5% (wt/vol) skimmed milk in tris-buffered saline (TBS)/0.1% (vol/vol) Tween-20 for 1 h at room temperature. The blots were incubated overnight at 4 °C with primary antibodies including N-terminus-cleaved GSD (ab215203) (Abcam, Cambridge) and beta-actin (12262), phospho-p65 NF-kB (3033), and phospho-Stat1 (9167) (Cell Signalling Technology Herts, UK) (1 : 1000 dilution in TBS, 5% milk). After washing in TBS/0.1% (vol/vol) Tween-20, the blots were incubated with HRP-conjugated secondary antibody at room temperature for 1 h in TBS/0.1% (vol/vol) Tween-20 and 5% milk. After the final washing, immunoreactivity was visualized using the chemiluminescent substrate ECL Plus (Bio-Rad, UK). A G-Box imaging system and Genesys software (Synoptics UK) were used to visualise the blots and a densitometry analysis was performed using Genetools (Synoptics UK). The level of cellular beta actin was used as a loading control.

### Inflammation antibody array

THP-1-derived macrophages were treated with glyco-FP20 derivatives (20 μM) or LPS (100 ng mL^−1^). The culture medium was collected after 18 h of incubation and analysed on a human inflammation array (Ray-Biotech, USA) containing 40 proinflammatory proteins to assess the relative levels of cytokine expression by the samples. The results are expressed as fold-increase relative to the control samples.

### ELISA

Human INFβ, IL1β, IL18, IL6 and MCP1 production was measured in cell culture medium using ELISA kits (R&D Systems, USA and Ray-Biotech, USA) following the manufacturer's instructions. In the final stage, absorbance was measured at 450 nm using a Sunrise (Tecan Group LTD., Switzerland) microplate reader. Protein concentration was calculated using GraphPad Prism version 7.01.

### Induction of pyroptosis in THP-1-derived macrophages

In this study, we show that similar to LPS, the glyco-FP20 derivatives specifically induced TLR4/MyD88/TRIF-dependent signalling, leading to M1 THP-1-derived macrophage polarisation. Further, we wanted to determine the ability of the glyco-FP20 derivatives to induce pyroptosis in human macrophages. For this purpose, we analysed their effect on the processing of gasdermin D (GSD) and production of IL-1β and IL-18 as biomarkers of pyroptosis. It is well known that LPS can induce pyroptosis in macrophages in the presence of a secondary signal such as ATP.^10^ To investigate the ability of the glyco-FP20 derivatives to induce pyroptosis, we utilised a cell model, where THP-1-derived macrophages were initially primed with LPS (100 ng mL^−1^) or glyco-FP20 derivatives (20 μM) (primary signal), and 3 h later treated with ATP (5 mM) (secondary signal). GSD processing in the cell lysates was measured at 6 h by western blotting. IL-1β and IL-18 were measured at 16 h by ELISA. To confirm that LPS or glyco-FP20 derivatives induced pyroptosis *via* GSD processing, we pretreated the THP-1-derived macrophages with a specific inhibitor of GSD processing U73 (Adipogen, UK) for 30 min prior to exposure to LPS or glyco-FP20 derivatives including ATP (as described above).

### P65 NF-kB and IRF3 nuclear translocation visualization using immunofluorescence analysis in THP-1-derived macrophages

THP-1-derived macrophages were seeded at a density of 5 × 10^4^ cells per well in PhenoPlate 96-well, black, optically clear flat-bottom, poly-d-lysine-coated microplates (PerkinElmer Inc., Milan, Italy), where they were exposed to 25 nM of PMA. After differentiation, the cell culture medium was replaced with either fresh RPMI (NT) or RPMI containing 100 ng mL^−1^ LPS or 20 μM FP20Rha, respectively. A time course treatment between 0 and 4 h was performed. After 4 h, the cells were fixed with 4% paraformaldehyde (Sigma-Aldrich, St. Louis, USA) and permeabilized with 0.5% Triton X-100 solution or fixed and permeabilized with 100% ice-cold methanol, according to the manufacturer's instructions. Afterwards, the cells were blocked using 1× PBS/5% BSA/0.3% Triton X-100. The cells were labeled with NF-κB p65 XP Rabbit mAb (1 : 400; 8242, CellSignaling Technology, Inc.) or phospho-IRF-3 (Ser386) XP Rabbit mAb (1 : 400; E7J8G, Cell Signaling Technology, Inc.). Then the cells were tagged with PhenoVue Fluor 568 conjugated anti-rabbit secondary antibody (2GXRB568C1, PerkinElmer Inc., Milan, Italy). Nuclei were counter-labelled with PhenoVue Hoechst 33342 Nuclear Stain (CP71, PerkinElmer Inc. Milan, Italy). Images were acquired using an Operetta CLS High-Content Analysis System and analyzed using the Harmony 4.5 software (PerkinElmer Inc., Milan, Italy).

### Flow cytometry

CD80 (BioLegend UK, B352986) expression on stimulated THP-1-derived macrophages was determined by flow cytometric analysis. The cell suspension was collected in Eppendorf tubes for analysis at different time points depending on the experimental design. The cells were centrifuged at 1300 rpm for 3 min, and the medium was removed from each sample. Secondly, PBS was used to wash cells before 150 μL trypsin was added to detach the cells. The cell pellets were washed with PBS and resuspended in 95 μL cell staining buffer (BioLegend, UK). Then 5 μL of Tru-Stain (BioLegend) blocking solution was added to each sample for 10 min for incubation at room temperature. After blocking, Bio Legend fluorescent PE-conjugated CD80 antibody was added in a 1 : 25 dilution for each sample before resting at 4 °C for 30 min in the dark. After incubation, the samples were washed twice with PBS. The cell pellets were resuspended in 100 μL PBS and transferred to 96-well plates for reading. 0.5 × 10^6^ events were read using an Agilent NovoCyte Advanteon VBR Flow Cytometer and analysed afterward using the FlowJo version 10.9.0 software.

### Statistical analysis

The results were analysed by one-way ANOVA, followed by a *post hoc* test (Tukey) for multiple comparisons using GraphPad Prism version 7.01. A value of *p* < 0.05 was considered significant.

## Results and discussion

### Glyco-FP20 derivatives induce TLR4/MyD88-dependent signalling pathways leading to M1 polarisation in THP-1-derived macrophages

**Fig. 2:**
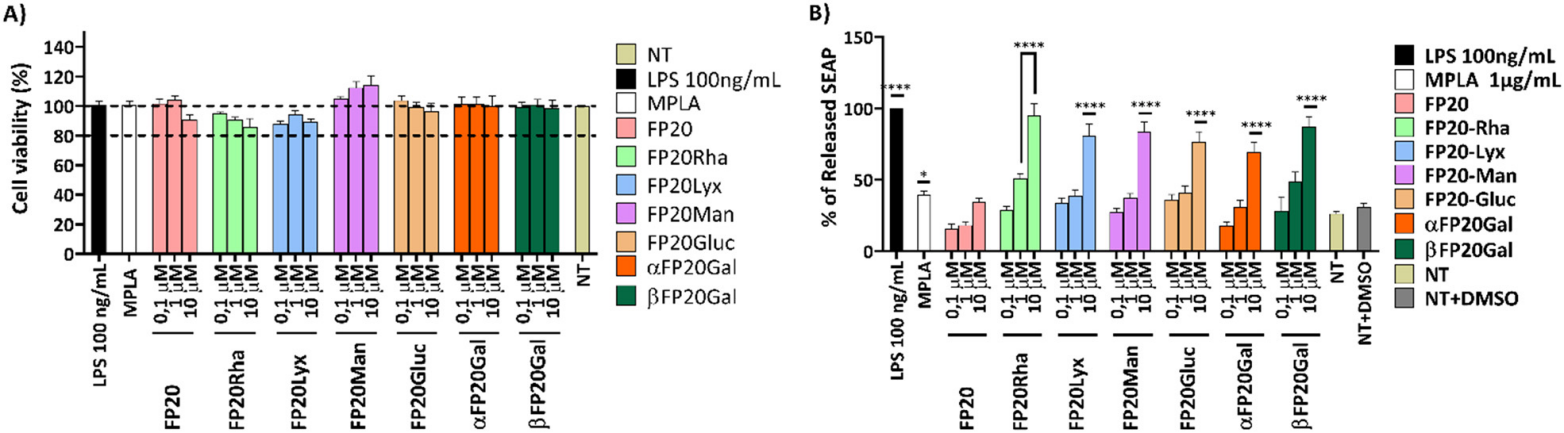
Cell viability (A) and activity of FP20 glycosylated derivatives (B) on THP-1-derived macrophages (TDM). TDM were treated with FP20 glycosylated derivatives: FP20, FP20Rha, FP20Lyx, FP20Man, FP20Gluc, αFP20Gal, βFP20Gal (0, 1–10 μM) for 16–18 hours. LPS (100 ng mL^−1^) and MPLA (1 μg mL^−1^) were used as controls. 100% viability was attributed to the controls NT (A) and to LPS (B). Cell viability was assessed using the MTT assay (A), and TDM activation was assessed using the SEAP assay (B). Data are expressed as mean ± SEM of at least three independent experiments (treated *vs.* nontreated: *p < 0.05; **p < 0.01; ***p < 0.001; ****p < 0.0001).

**Fig. 3:**
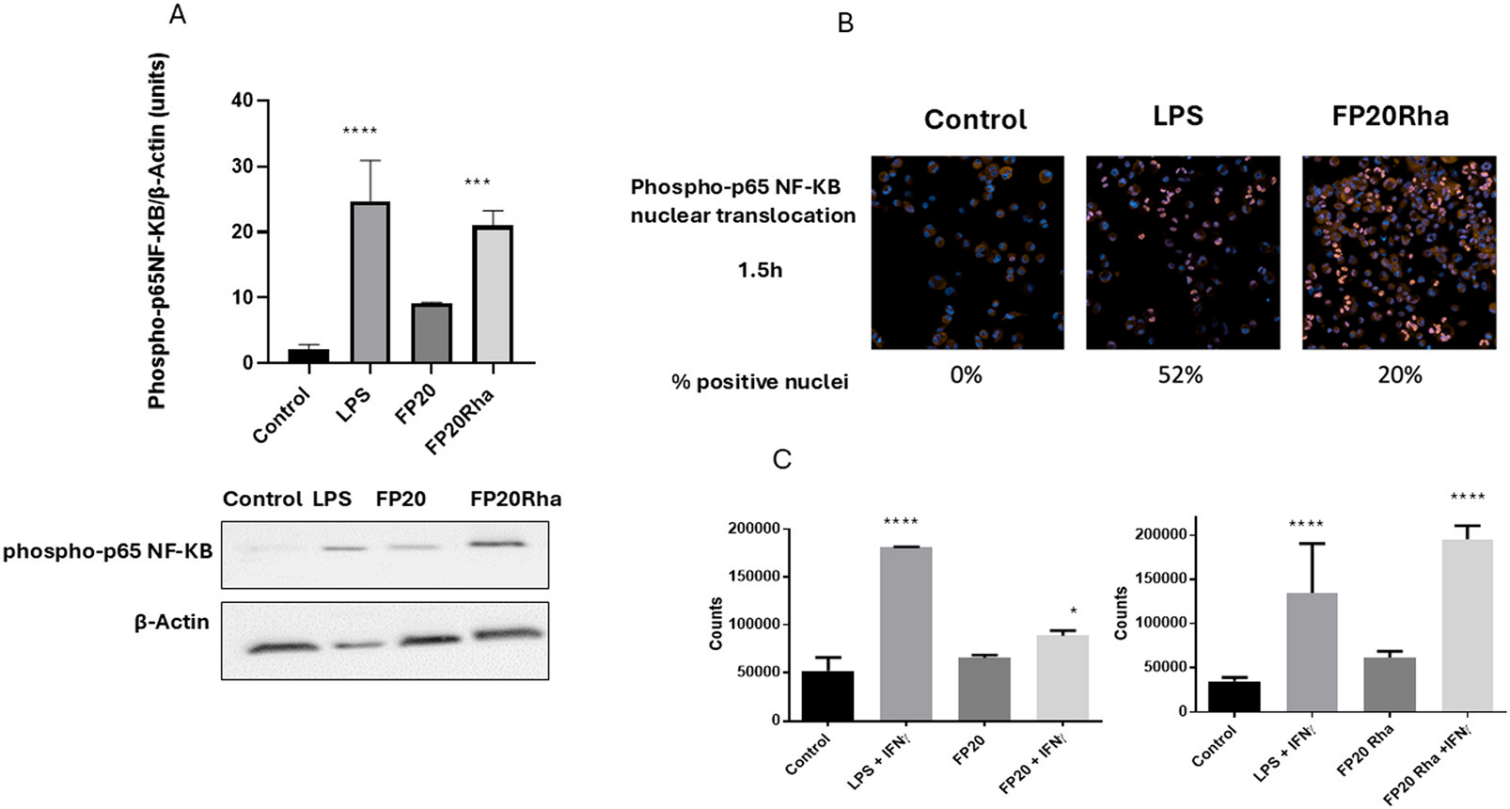
FP20 derivatives induce TLR4/MyD88/p65 NF-kB signaling and CD80 expression in THP-1-derived macrophages. A. THP-1-derived macrophages were treated with FP20 derivatives (FP20 and FP20 Rha at 20 μM) and LPS (100 ng ml^−1^) for 1 hour. Cell lysates were collected and analysed for phospho-p65 NF-kB expression and normalized to β-actin. B. THP-1-derived macrophages were treated with FP20 Rha at 20 μM and LPS (100 ng ml^−1^) for 1.5 hours and stained with phospho p65 NF-kB antibody. C. THP-1-derived macrophages were treated with FP20 and FP20 Rha at 20 μM and LPS (100 ng ml^−1^) in the presence of IFNγ (20 ng ml^−1^) for 24 hours. Results are from three (A), one (B) and two (C) independent experiments. Statistically significant results are indicated as *p < 0.05 and ****p < 0.0001 *versus* control.

The selectivity of the new glyco-FP20 derivatives towards TLR4 was tested using HEK-Blue cells (5). To study the functional potential of the glyco-FP20 derivatives on MyD88-dependent TLR4 signalling, THP-1-derived human macrophages were used as an *in vitro* cell model (Fig. 2). The experimental design was based on two readouts, *i.e.*, the activation of TLR4 second messengers (NF-kB phosphorylation/translocation) and the production of TLR4-dependent pro-inflammatory proteins associated with this pathway. Additionally, we compared the potential of the LPS-driven TLR4/MyD88 pathways with the glyco-FP20 derivatives. The results showed that similar to LPS, FP20, FP20 Lyc and FP20 Rha induced p65 NF-kB phosphorylation and translocation to the nucleus including IkB alpha degradation (data not shown) in THP-1-derived macrophages (Fig. 3A and B). Furthermore, results demonstrated the ability of FP20 Rha, and to a lesser extent FP20 to potentiate LPS/IFNγ-induced CD80 expression (important M1 macrophage biomarker). Intriguingly, the effect of FP20 Rha on CD80 expression was stronger than the parent FP20 molecule (Fig. 3C).

Subsequently, we examined the potential of the glyco-FP20 derivatives to mediate the production of TLR4-dependent proinflammatory markers of M1 polarisation. For this purpose, we utilised an inflammation antibody array to measure the production of 40 proinflammatory proteins. The antibody array results revealed that similar to LPS, all the glyco-FP20 derivatives induced the release of 24/40 TLR4-dependent proinflammatory proteins (Table 1). The amplitude of production of different proinflammatory mediators varied among the glyco-FP20 derivatives and we validated the most upregulated proteins (IL-1β, IL-6 and MCP-1). The ELISA results showed that FP20, FP20 Lyx and FP20 Rha upregulated IL-1β (Fig. S1, ESI†), IL-6 (Fig. S2, ESI†), and MCP-1 (Fig. S3, ESI†) in a dose-dependent manner in THP-1-derived macrophages. These results clearly showed the potential of the glyco-FP20 derivatives to induce TLR4-dependent signalling associated with M1 polarisation in THP-1-derived macrophages.

**Table 1:** FP20 derivatives upregulate the release of TLR4-dependent proinflammatory mediators in THP-1-derived macrophages. THP-1-derived macrophages were treated with LPS (100 ng ml^−1^) and FP20 derivatives (20 μM) for 24 hours. Cell media were analysed using an antibody inflammation array containing 40 proinflammatory proteins. Semi-quantitative results are normalized to untreated control cells (fold increase)

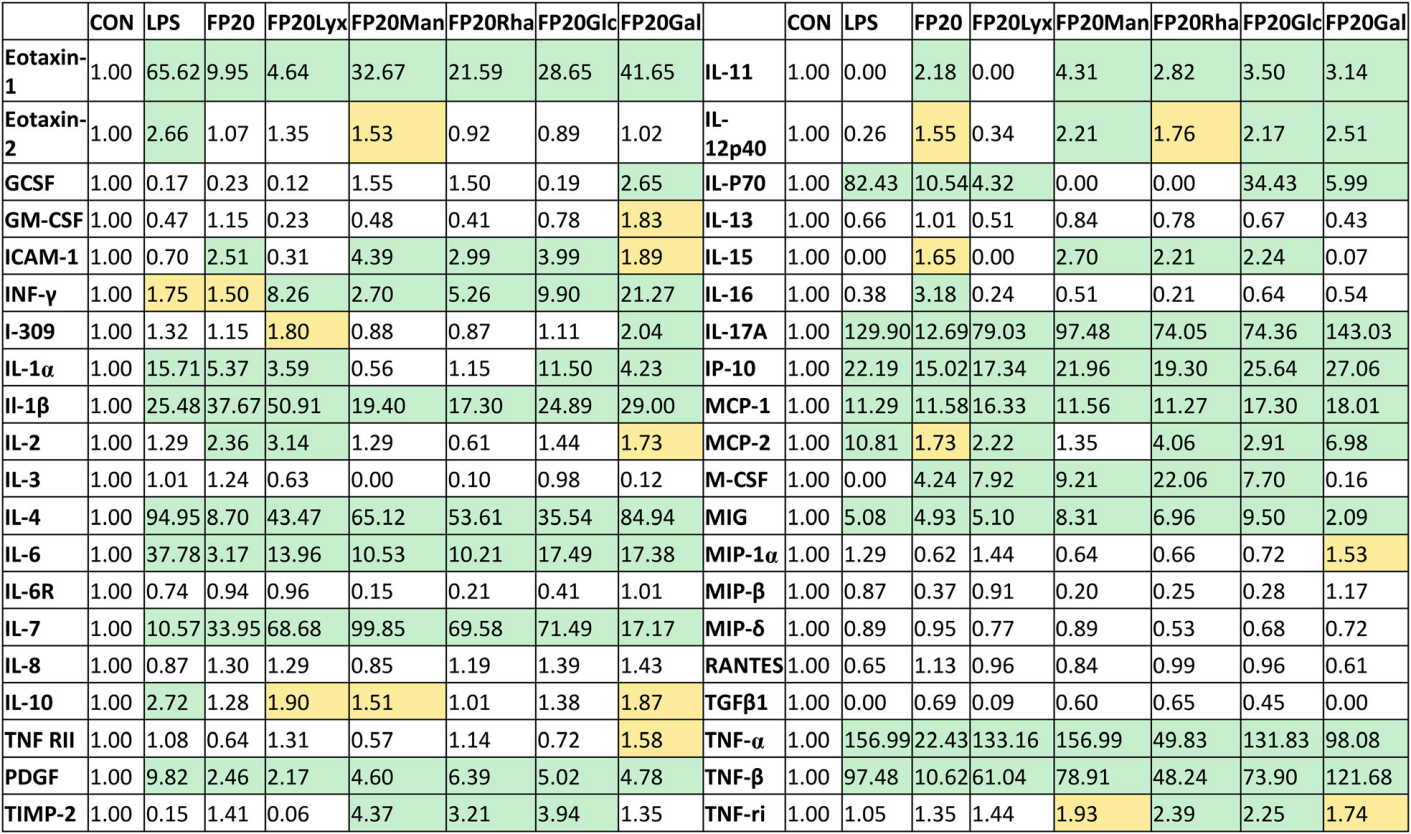

### Effect of glyco-FP20 derivatives on TLR4/TRIF-dependent signalling pathways in THP-1-derived macrophages

**Fig. 4:**
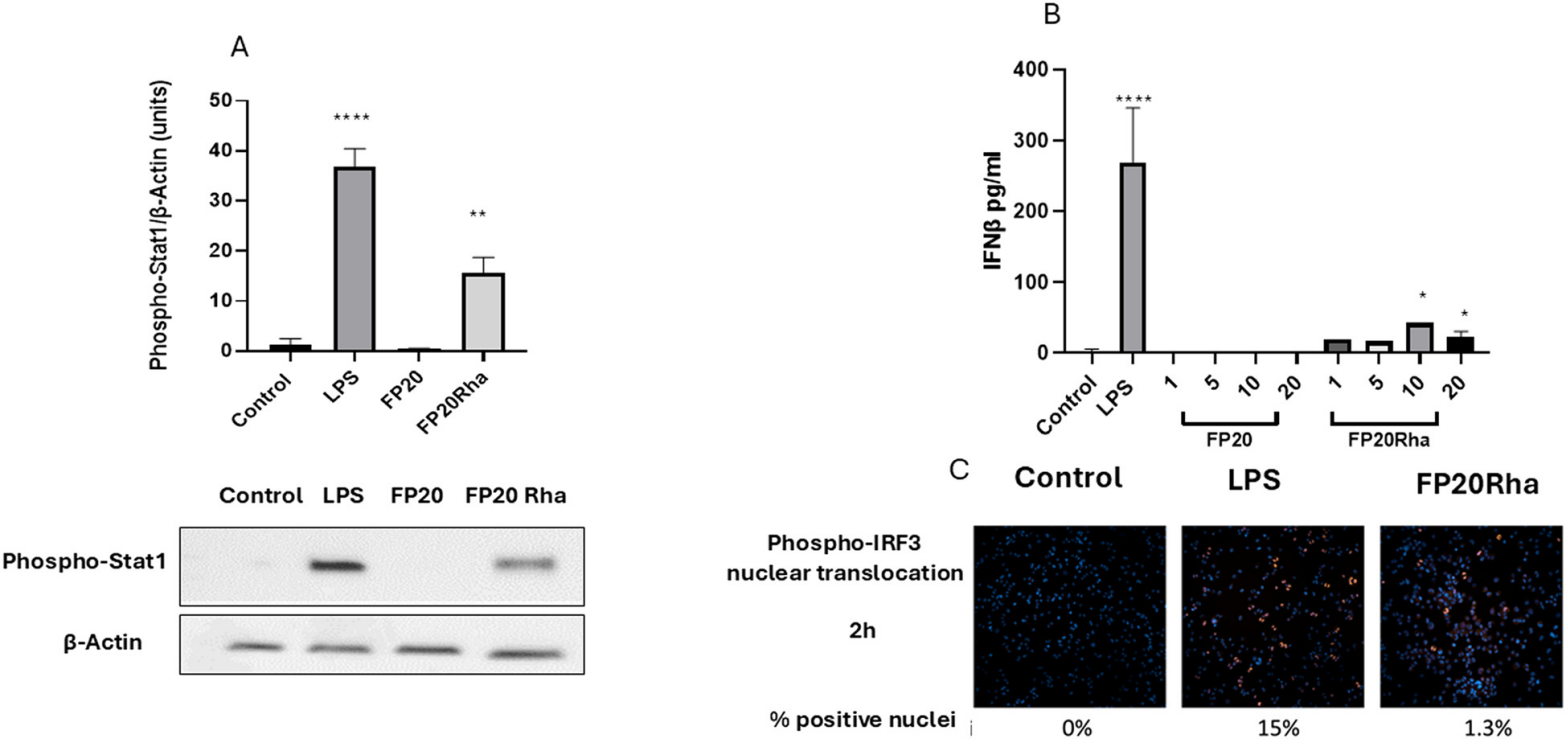
FP20 derivatives induce TLR4/TRIF/STAT1/IRF3 signaling in THP-1-derived macrophages. THP-1-derived macrophages were treated with FP20 derivatives (20 μM) and LPS (100 ng ml^−1^) for 3 hours. Cell lysates were analysed for phospho-STAT1 expression and normalised to actin by western blotting. B. THP-1-derived macrophages were treated with FP20 Rha at 20 μM and LPS (100 ng ml^−1^) for 2 hours and stained with phospho-IRF3 antibody. C. THP-1-derived macrophages were treated with FP20 and FP20 Rha at 20 μM and LPS (100 ng ml^−1^) for 6 hours, and the medium was analysed for IFNβ released by ELISA. Results are from three (A and B) independent experiments. Statistically significant results are indicated as *p < 0.05, **p < 0.01, ***p < 0.001, ****p < 0.0001 *versus* control.

The effects of the glyco-FP20 derivatives on the alternative TRIF-dependent TLR4 signalling pathway were investigated. For this purpose, we analysed their effect on STAT1 phosphorylation and phospho-IRF-3 nuclear translocation as second messengers (first readout) in this pathway. The results from western blotting and immunostaining analyses showed that only FP20 Rha significantly induced STAT1 phosphorylation and phospho-IRF3 nuclear translocation compared to the parent FP20 molecule (Fig. 4A and B).

Previously we showed that the short time of LPS-induced IFNβ availability may be associated with the signalling properties of this molecule through interferon receptor alpha/beta (IFNAR).^11^ Following this, experiments monitoring IFNβ (second readout) production were considered after 6 h of LPS and glyco-FP20 derivative exposure. Again, the ELISA results showed that only FP20 Rha significantly induced IFNβ production compared to parent molecule FP20 in THP-1-derived macrophages (Fig. 4C). These data revealed that glycosylation of FP20 Rha displayed potential as a lead compound to activate both MyD88 and TRIF TLR4-dependent signalling in THP-1-derived macrophages.

### Glyco-FP20 derivatives induced pyroptosis in THP-1-derived macrophages

In this study we showed that similar to LPS, the glyco-FP20 derivatives specifically induced TLR4/MyD88/TRIF-dependent signalling, leading to M1 THP-1 macrophage polarisation. Further, we wanted to determine the ability of the glyco-FP20 derivatives to induce pyroptosis in human macrophages. For this purpose, we analysed their effect on GSD processing and production of IL-1β and IL-18 as biomarkers of pyroptosis. To investigate the ability of the glyco-FP20 derivatives to induce pyroptosis, we utilised a cell model, where THP-1-derived macrophages were initially primed with LPS or glyco-FP20 derivatives (primary signal), and 3 h later treated with ATP (secondary signal).

**Fig. 5:**
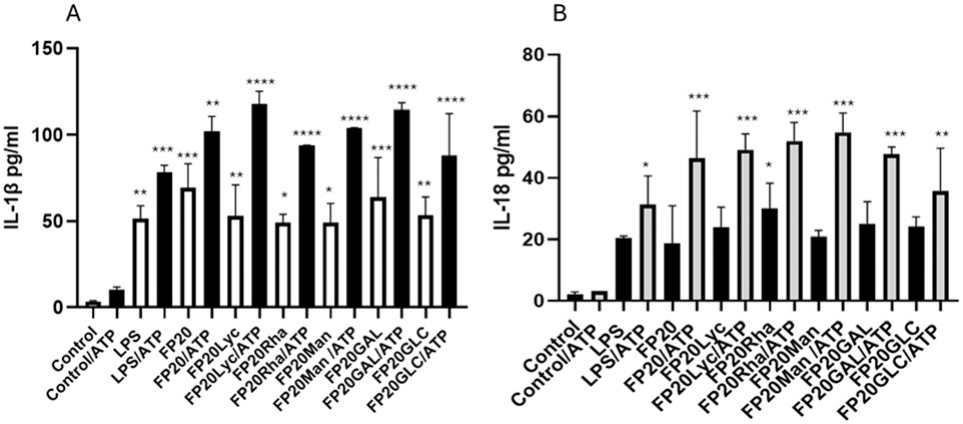
THP-1-derived macrophages were exposed to FP20 derivatives (20 μM) and LPS (100 ng ml^−1^). ATP (5 mM) was added after 3 hours, and cell media were collected after 16 hours and analysed for IL-1β (A) and IL-18 (B) by ELISA. Results are from 3 independent samples. **p < 0.01, ANOVA in the presence or absence of ATP.

**Fig. 6:**
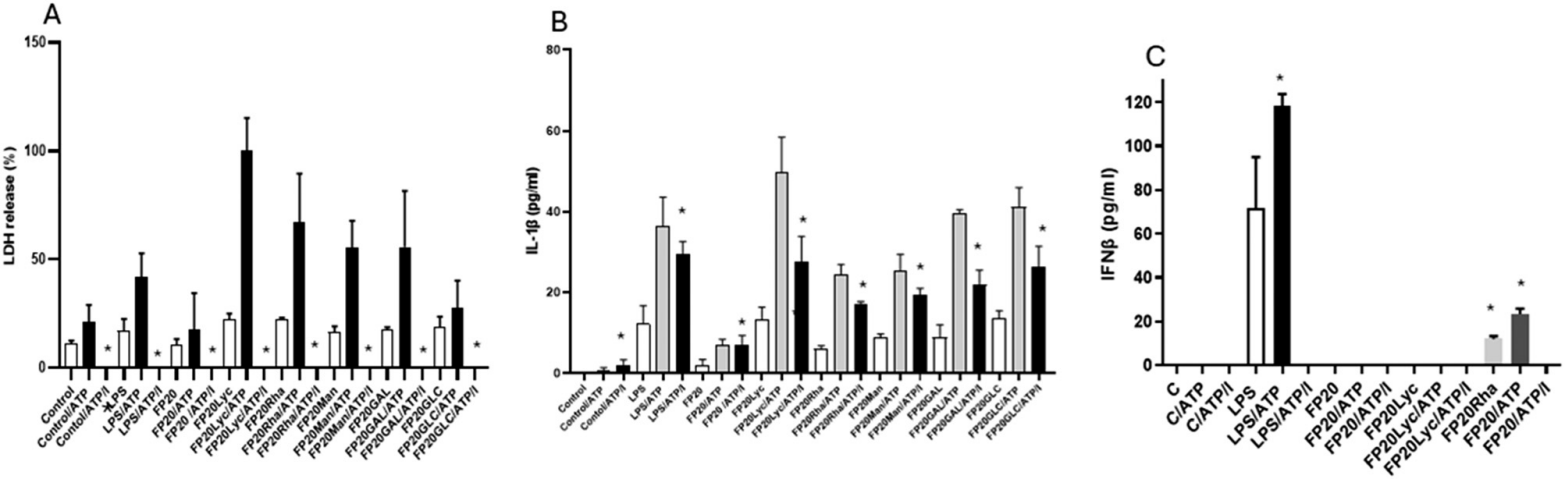
THP-1-derived macrophages were pre-treated with U73 (10 μM) for 30 min before exposure to FP20 derivatives (20 μM) and LPS (100 ng ml^−1^). ATP (5 mM) was added after 3 hours, and cell media were collected after 16 hours (A and B) and 6 hours, (C) and analysed for LDH (A), IL-1β (B) and IFN-β release by ELISA. Results are from 3 independent samples. *p < 0.05 ***p < 0.001, ANOVA in the presence or absence of ATP.

In the first series of experiments, we monitored the contribution of ATP as a second signal in the release of IL-1β and IL-18 in the THP-1 macrophage cell model. The ELISA results demonstrated that similar to LPS, all 6 glyco-FP20 derivatives induced TLR4/My88 pathways, leading to IL-1β and IL-18 release, which was significantly potentiated in the presence of ATP (Fig. 5A and B and S5†). Similarly, ATP potentiated the TLR4/TRIF pathways, leading to the release of INFβ; however, only LPS and FP20 Rha displayed a positive impact on these signalling pathways (Fig. 6C). These results showed that ATP as a second signal is important to increase the amplitude of FP20 derivative-induced TLR4/MyD88/TRIF dependent pyroptosis in THP-1-derived macrophages. In the second series of experiments, we examined the ability of the glyco-FP20 derivatives to induce N-terminus processing of GSD (biomarker of pyroptosis), which is associated with opening of plasmatic membrane pores, leading to the release of proinflammatory proteins including IL-1β and IL-18 (biomarkers of pyroptosis). For this purpose, we used a specific GSD inhibitor (U73, Adipogen, UK), which was shown to block the N-terminus processing of GSD.^12^ The results from the western blotting analysis showed that LPS and the glyco-FP20 derivatives induced N-terminus processing of GSD, and U73 significantly blocked GSD conversion (Fig. S4†). Interestingly, we found that both LPS and the glyco-FP20 derivatives were potent to induce GSD processing in the absence of a second trigger such as ATP. This result is linked to our previous finding, demonstrating that LPS and glyco-FP20 derivatives can induce IL-1β and IL-18 in a dose-dependent manner in the absence of ATP (Table 1 and Fig. S3 and S5 ESI†).

Finally, we investigated the role of blocking GSD processing by using the U73 inhibitor on THP-1 macrophage viability and the release of IL-1β and INFβ. The results from LDH ELISA showed that U73 completely blocked LPS and FP20 derivative-induced LDH release (Fig. 6A) and LPS and FP20 Rha induced IFNβ production (Fig. 6C), and partially decreased IL-1β release (Fig. 6B) in THP-1-derived macrophages.

In summary, the results from this section show that all 5 glyco-FP20 derivatives induced TLR4/MyD88-dependent pyroptosis in THP-1-derived macrophages in the absence of a second signal such as ATP. We further showed that the potential of FP20 Rha to operate *via* TLR4/TRIF signalling pathways, leading to the production of IFNβ. ATP potentiated the amplitude of pyroptosis based on the release of IL-1β, IL-18 and LDH. Importantly, blocking GSD processing led to a significant decrease in pyroptosis, suggesting the important role of GSD in glyco-FP20 compound-induced pyroptosis in THP-1-derived macrophages.

## Conclusions

Modulation of the TLR4 signalling pathways has been documented as a critical event in a variety of inflammatory-related responses including vaccine adjuvant development. Recently, we published a new series of TLR4 agonists called FP20, which are promising vaccine adjuvant candidates.^6^ We generated five glyco-FP20 derivatives and showed that glycosylation of the free C-6 hydroxyl of the FP20 parent molecule increased its potency, which is related to the improvement in the interaction with TLR4 and changes in the physico-chemical properties of the FP20 derivatives.^8,9^

The main goal of this study was to investigate the molecular mechanisms by which these FP20 molecules affect TLR4 signalling in the context of inflammatory responses in human THP-1-derived macrophages. We selected a human macrophage model cell system as a biosensor because it can change its phenotype to perform differential activities in different phases of vaccine adjuvant specific immune responses. Polarised macrophages are broadly classified into two groups, *i.e.*, classical activated M1 (pro-inflammatory) and alternative-activated M2 (anti-inflammatory). The M1 macrophage phenotype is characterized by the production of high levels of pro-inflammatory mediators and reactive nitrogen and oxygen intermediates, and promotion of Th1 responses essential for vaccine adjuvants specific immune responses. In this context, TLR4 signalling plays an essential role in M1 macrophage polarisation, which induces secretion of large amounts of cytokines such as IL-1β, tumour necrosis factor alpha (TNFα), IL-6, IL-8 and IL-23. Additionally, M1 produces the Th1 cell-attracting chemokines CXCL9 and CXCL10, which are associated with antigen-specific Th1 and Th17 cell inflammatory responses.^13^ The application of the macrophage polarisation model system has been proven to be suitable for the analysis and drug discovery research for screening novel immunomodulators. An *in vitro* macrophage-based approach has been successfully used to evaluate the immunomodulatory effects of approved drugs and novel molecules.^14,15^ In this context, pharmacological activation of TLR4, which is associated with M1 macrophages, is beneficial for the generation of novel adjuvant-based vaccines.

Canonical and noncanonical inflammasome activity is critical for innate immune responses to LPS-triggered TLR4 signalling.^16^ The canonical inflammasome activates caspase-1, whereas noncanonical inflammasomes activate caspase-4/5. Subsequently, LPS-activated caspase-1/4/5 cleaves the substrate GSD to generate a GSD-cleaved fragment that forms membrane pores. GSD-associated pores trigger plasma membrane rupture and pyroptosis.^17,18^ Also, GSD pores trigger ionic flux, leading to active NLRP3 inflammasomes, and consequently generating active caspase-1, which cleaves pro-IL-1β and pro-IL-18 released through the membrane pores.^19,20^

In this study, we determined the ability of glyco-FP20 derivatives to induce pyroptosis in THP-1-derived macrophages. For this purpose, ATP was used as a second promo signal to induce pyroptosis. We showed that similar to LPS, all 5 glyco-FP20 compounds tested induced LDH/GSD processing-dependent pyroptosis in an ATP-independent manner in THP-1-derived macrophages. Intriguingly, the presence of ATP as a secondary signal potentiated the level of LDH/IL-1β/IL-18 release, suggesting the important role of the second signal in the amplification of the immune response. This also suggested that similar to LPS, the FP20 derivatives may induce both canonical and non-canonical pathways, leading to the induction of pyroptosis in THP-1-derived macrophages; however, further studies are required.

Importantly, our study showed the potential of FP20 Rha, not any of the other glyco-FP20 derivatives or the parent compound FP20, to operate *via* TLR4/TRIF signalling pathways, leading to the production of interferons. This also show that similar to LPS, FP20 Rha could induce translocation of TLR4 to endosomes to initiate TRIF/STAT1/IRF3/INFβ signalling pathways. Also, the vaccine adjuvant MPLA activates both the MyD88 and TRIF pathways with a bias towards TRIF.^4^ This important finding suggests that FP20 Rha can be selected as a lead compound in conjugate vaccine generation, where the production of interferons is essential for the immune response.

## Data availability

The data supporting this article have been included as part of the ESI.†

## Author contributions

ARF: investigation, methodology, writing – review and editing; ZA: investigation, methodology; AR: investigation, methodology, writing – review and editing; AI, FL, MMS, NS, VA: investigation, methodology; GP: conceptualization, funding acquisition, investigation, methodology, project administration, writing – original draft, writing – review and editing; FP: conceptualization, resources, supervision, funding acquisition, methodology, project administration, validation, writing – original draft, writing – review and editing.

## Conflicts of interest

There are no conflicts to declare.

## Supplementary Material

MD-016-D4MD00950A-s001
