# Supplementary material for: Towards more efficient synthetic immunomodulators: biological characterization and mechanism of action of monosaccharide-derived TLR4 agonists
Source: RSC Med Chem. 2025 Feb 18;16(5):2261–9. doi: 10.1039/d4md00950a (PMC11926738; doi:10.1039/d4md00950a)
Supplement: MD-016-D4MD00950A-s001 [file MD-016-D4MD00950A-s001.pdf]

Supplementary Information (SI) for RSC Medicinal Chemistry.
This journal is © The Royal Society of Chemistry 2025

Supplementary Information

Towards more efficient synthetic immunomodulators: biological characterization and mechanism of action of monosaccharide-derived TLR4 agonists

Ana Rita Franco[a]†, Zaineh Aladailleh[b]† Alessio Romerio[a], , Alice Italia[a], Fedrico Lami[a,] Monsoor Shaik[a], Natalia Skupinska[b], Valentina Artusa[a], Grisha Pirianov[b] and Francesco Peri*[a]

Table of contents

Figure S 1 FP20 derivatives induce IL-1β release in THP-1 macrophages. 2

Figure S 2 FP20 derivatives induce IL-6 release in THP-1 macrophages. 2

Figure S 3 FP20 derivatives induce MCP-1 release in THP-1 macrophages...........................3

Figure S 4 FP20 derivatives induce pGSD release in THP-1 macrophages. 3

Figure S 5 FP20 derivatives induce IL-18 release in THP-1 macrophages. 4

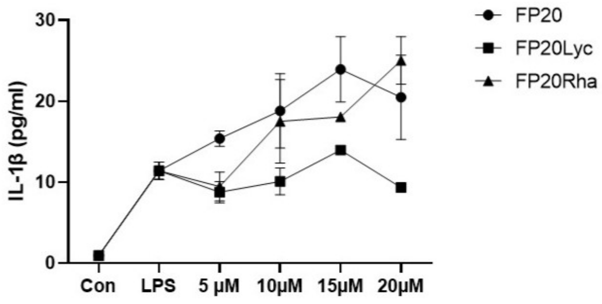

IL-1β(pg/ml)	LPS	5µM	10µM	15µM	20µM
FP20	P<0.05	P<0.01	P<0.001	P<0.001	P<0.001
FP20Lyc	P<0.0001	P<0.001	P<0.0001	P<0.0001	P<0.001
FP20Rha	P<0.01	P<0.01	P<0.001	P<0.001	P<0.0001

Figure S 1 FP20 derivatives induce IL-1b release in THP-1 macrophages.

THP-1 macrophages were treated with FP20 and FP20 derivatives (FP20Lyx and FP20Rha) with concentration ranging (0-20 µM) and LPS (100 ng/ml) for 24 hours. Cell mediums were analysed for IL-1β release by ELISA. Results are from three independent experiments, respectively. Statistically significant results are indicated as *p<0.05, **p<0.01, ***p<0.001, ****p<0.0001 versus control (table).

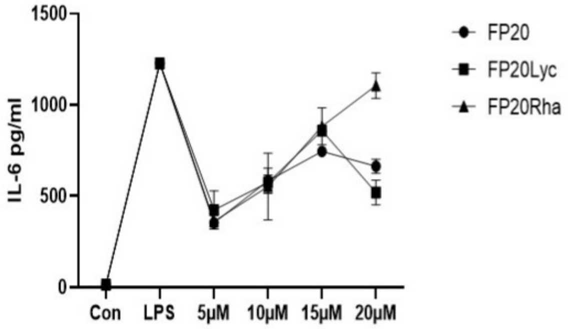

IL-6 pg/ml	LPS	5µM	10µM	15µM	20µM
FP20	P<0.0001	P<0.001	P<0.0001	P<0.0001	P<0.0001
FP20Lyc	P<0.0001	P<0.01	P<0.001	P<0.001	P<0.01
FP20Rha	P<0.0001	NS	P<0.05	P<0.01	P<0.001

Figure S 2 FP20 derivatives induce IL-6 release in THP-1 macrophages.

THP-1 macrophages were treated with FP20 and FP20 derivates (FP20Lyx and FP20Rha) with concentration ranging (0-20 µM) and LPS (100ng/ml) for 24 hours. Cell mediums were analysed for IL-6 release by ELISA. Results are from three independent experiments, respectively. Statistically significant results are indicated as *p<0.05, **p<0.01, ***p<0.001, ****p<0.0001 versus control.

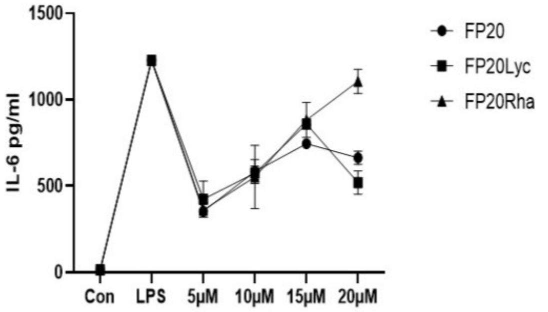

IL-6 pg/ml	LPS	5μM	10μM	15μM	20μM
FP20	P<0.0001	P<0.001	P<0.0001	P<0.0001	P<0.0001
FP20Lyc	P<0.0001	P<0.01	P<0.001	P<0.001	P<0.01
FP20Rha	P<0.0001	NS	P<0.05	P<0.01	P<0.001

Figure S 3 FP20 derivatives induce MCP-1 release in THP-1 macrophages.

THP-1 macrophages were treated with FP20 and FP20 derivates (Lyc and Rha) with concentration ranging (0-20 μM) and LPS (100ng/ml) for 24 hours. Cell mediums were analysed for MCP-1 release by ELISA. Results are from three independent experiments, respectively. Statistically significant results are indicated as *p<0.05, **p<0.01, ***p<0.001, ****p<0.0001 versus control (table).

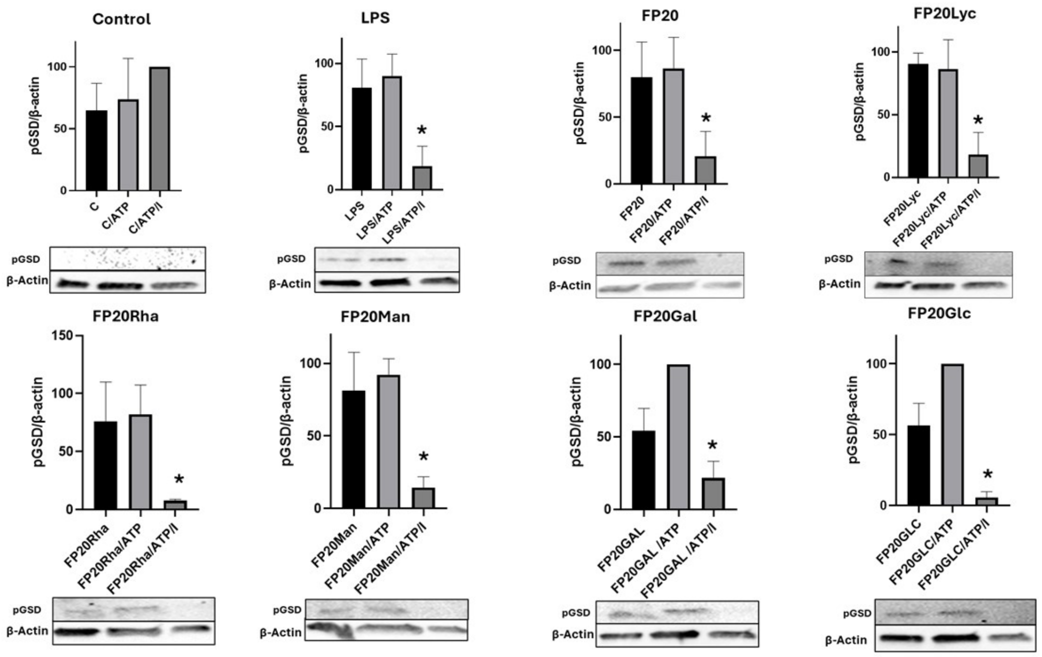

Figure S 4 FP20 derivatives induce pGSD release in THP-1 macrophages.

THP-1 macrophages were pre-treated with U73 (10 μM) for 30 mins before exposure to FP20 derivatives (20 μM) and LPS (100ng/ml). ATP (5mM) was added after 3 hours, and cell lysates were isolated after 6 hours. Cell lysates were analysed for Gasdermin D processing (pGSD) and normalised to actin. Results are from three independent experiments. *p<0.05 Anova in the presence or absence of U73 (I).

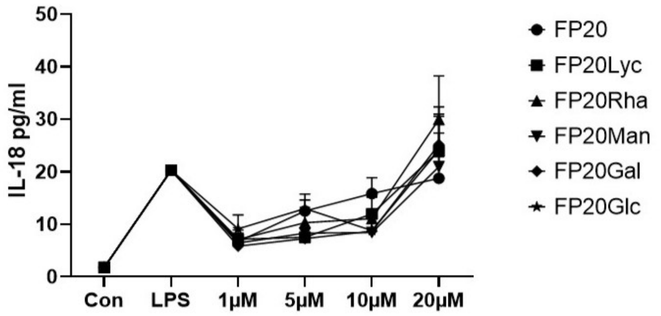

IL-18 pg/ml	LPS	1µM	5µM	10µM	20µM
FP20	P<0.01	NS	NS	P<0.05	P<0.01
FP20Lyc	P<0.0001	NS	NS	P<0.001	P<0.0001
FP20Rha	P<0.001	NS	P<0.05	P<0.05	P<0.001
FP20Man	P<0.0001	P<0.05	P<0.01	P<0.01	P<0.0001
FP20Gal	P<0.001	NS	NS	NS	P<0.0001
FP20Glc	P<0.0001	P<0.05	P<0.01	P<0.05	P<0.0001

Figure S 5 FP20 derivatives induce IL-18 release in THP-1 macrophages.

THP-1 macrophages were treated with FP20 and FP20 derivates (FP20Lyx, FP20Rha, FP20Man, FP20Gal and FP20Glc) with concentration ranging (0-20 µM) and LPS (100ng/ml) for 24 hours. Cell mediums were analysed for IL-18 release by ELISA. Results are from three independent experiments, respectively. Statistically significant results are indicated as *p<0.05, **p<0.01, ***p<0.001, ****p<0.0001 versus control (table).
